# Transient and steady-state selection in the striatal microcircuit

**DOI:** 10.3389/fncom.2013.00192

**Published:** 2014-01-20

**Authors:** Adam Tomkins, Eleni Vasilaki, Christian Beste, Kevin Gurney, Mark D. Humphries

**Affiliations:** ^1^Department of Computer Science, University of SheffieldSheffield, UK; ^2^INSIGNEO Institute for in Silico Medicine, University of SheffieldSheffield, UK; ^3^Cognitive Neurophysiology, Universitätsklinikum Carl Gustav CarusTU Dresden, Germany; ^4^Adaptive Behaviour Research Group, Department of Psychology, University of SheffieldSheffield, UK; ^5^Faculty of Life Sciences, University of ManchesterManchester, UK

**Keywords:** response selection, action selection, striatum, Huntington's disease, basal ganglia, excitotoxicity

## Abstract

Although the basal ganglia have been widely studied and implicated in signal processing and action selection, little information is known about the active role the striatal microcircuit plays in action selection in the basal ganglia-thalamo-cortical loops. To address this knowledge gap we use a large scale three dimensional spiking model of the striatum, combined with a rate coded model of the basal ganglia-thalamo-cortical loop, to asses the computational role the striatum plays in action selection. We identify a robust transient phenomena generated by the striatal microcircuit, which temporarily enhances the difference between two competing cortical inputs. We show that this transient is sufficient to modulate decision making in the basal ganglia-thalamo-cortical circuit. We also find that the transient selection originates from a novel adaptation effect in single striatal projection neurons, which is amenable to experimental testing. Finally, we compared transient selection with models implementing classical steady-state selection. We challenged both forms of model to account for recent reports of paradoxically enhanced response selection in Huntington's disease patients. We found that steady-state selection was uniformly impaired under all simulated Huntington's conditions, but transient selection was enhanced given a sufficient Huntington's-like increase in NMDA receptor sensitivity. Thus our models provide an intriguing hypothesis for the mechanisms underlying the paradoxical cognitive improvements in manifest Huntington's patients.

## 1. Introduction

Finding the neural substrate for the process of “selection” is key to furthering our understanding of decision-making (Ding and Gold, 2013), action selection (Mink, 1996; Grillner et al., 2005), planning (Houk and Wise, 1995), action sequencing (Jin and Costa, 2010), and even working memory (Gruber et al., 2006). A unifying proposal is that the basal ganglia forms just such a generic selection mechanism (Prescott et al., 1999; Redgrave et al., 1999); this proposal neatly explains why the basal ganglia have been hypothesized to contribute to each of these functions. But specifying the computational process of selection by the basal ganglia is challenging (Berns and Sejnowski, 1998; Gurney et al., 2001a,b; Humphries et al., 2006; Leblois et al., 2006).

A particular unknown is the computational role of the basal ganglia's input nucleus, the striatum. The striatum's GABAergic projection neurons comprise the vast majority of cells and are connected by local collaterals of their axons (Wilson and Groves, 1980). The lack of layers or of clear axial preferences in the direction of dendrites or axons suggests that striatal tissue is homogeneous in all three dimensions (Humphries et al., 2010). Such GABAergic connectivity naturally lends itself to the idea that the striatum forms a vast recurrent network that, locally, implements a winner-takes-all computation (Alexander and Wickens, 1993; Fukai and Tanaka, 1997; Wickens, 1997). The weak strength of synapses between the projection neurons (Jaeger et al., 1994; Czubayko and Plenz, 2002; Tunstall et al., 2002) is difficult to reconcile with this proposal (Plenz, 2003), as they suggest projection neuron output can only modulate ongoing activity and not outright inhibit their targets.

Here we report an alternative, transient form of selection that can occur in weak, sparse networks of striatal projection neurons. Using our three-dimensional network model of distance-dependent connections in the striatal microcircuit (Humphries et al., 2009b, 2010), we explored the effect on striatal output of competing inputs to two projection neuron populations. We found that rapidly stepped input to one population caused a transient competitive effect on the two populations' outputs, which disappeared after around 100 ms. In response to the same inputs, we also found that sufficiently dense striatal connectivity could result in steady-state competition, where the post-step equilibrium activity of each population reflects the inhibition of one by the other.

To compare transient and steady-state selection we challenged both forms of model to account for the paradoxical response selection results of Beste et al. (2008). They found that manifest Huntington's disease patients were both faster and less error prone than controls on a simple two-choice reaction-time task. As Huntington's disease primarily results in striatal damage, this suggests the hypothesis that changes in the striatum directly affect response selection. We expand on the role of the striatum in signal selection, by describing a framework for signal selection that may account for both the typical decline in performance for most tasks under Huntington's conditions Ho et al. (2003), as well as a mechanism for increased performance under the same conditions. We thus explored how Huntington's disease-like changes to our striatum models could affect both transient and steady-state selection, and sought whether the effect on either form of selection could explain the results of Beste et al. (2008), while also accounting for the usual cognitive impairment in Huntington's disease (Lawrence et al., 1998; Ho et al., 2003).

## 2. Materials and methods

We study here an updated version of our prior, full-scale model of striatum (Humphries et al., 2009b, 2010). Compared to those models, the model here brings together the three-dimensional anatomy model from Humphries et al. (2010) with an updated version of the dopamine-modulated projection neuron model from Humphries et al. (2009a).

### 2.1. Spiking neuron models

The basic model neuron used in the large scale striatal model is derived from the model neuron proposed in Izhikevich (2003), which was extended to encompass the effects of dopamine modulation on intrinsic ion channels and synaptic input in Humphries et al. (2009b).

In the biophysical form of the Izhikevich model neuron, *v* is the membrane potential and the “recovery variable” *u* is the contribution of the neuron class's dominant ion channel:

(1) $$C\dot{v}=k\left(v-v_{r}\right)\left(v-v_{t}\right)-u+I$$

(2) $$\dot{u}=a\left[b\left(v-v_{r}\right)-u\right]$$

with reset condition

$$\text{if }v>v_{\text{peak}}\text{ then }v\leftarrow c,\;u\leftarrow u+d$$

where in the equation for the membrane potential (Equation 1), *C* is capacitance, *v*_*r*_ and *v*_*t*_ are the resting and threshold potentials, *I* is a current source, and *c* is the reset potential. Parameter *a* is a time constant governing the time scale of the recovery due to the dominant ion channel. Parameters *k* and *b* are derived from the I-V curve of the target neuron behavior, where *b* describes how sensitive the recovery variable *u* is to fluctuations in the membrane potential *v*. Parameter *d* describes the after spike reset of recovery variable *u*, and can be tuned to modify the rate of spiking output.

#### 2.1.1. Projection neuron model

The projection neuron models' parameter values and their source are given in Table 1. Parameters *C*, *d*, *v*_*t*_, and the AMPA synaptic conductance *g*_ampa_ (see below) were found by searching for the best-fit to the f-I curve and spiking input–output functions of the Moyer et al. (2007) 189-compartment projection neuron model (Humphries et al., 2009a).

**Table 1:** **Intrinsic parameters for the projection model**.

**Parameter**	**Value**	**Source**
*a*	0.01	Mahon, 2000; Izhikevich, 2007b
*b*	−20	Izhikevich, 2007b
*c*	−55 mV	Izhikevich, 2007b
*k*	1	Izhikevich, 2007b
*v*_*r*_	−80 mV	Izhikevich, 2007b
*v*_peak_	40 mV	Izhikevich, 2007b
*C*	15 pF	Humphries et al., 2009a
*v*_*t*_	−30 mV	Humphries et al., 2009a
*d*	91	Humphries et al., 2009a
*K*	0.0289	Humphries et al., 2009a
*L*	0.331	Humphries et al., 2009a
α	0.032	Humphries et al., 2009a

In Humphries et al. (2009a) we showed how this model can capture key dynamical phenomena of the projection neuron: the slow-rise to first spike following current injection; paired-pulse facilitation lasting hundreds of milliseconds; and bimodal membrane behavior emulating up- and down-state activity under anaesthesia and in stimulated slice preparations.

#### 2.1.2. Fast-spiking interneuron model

For the FSI model, Equation (2) for the *u* term is given by (Izhikevich, 2007b)

(3) $${\dot{u}}_{fs}=\{\begin{array}{ll} -au_{fs} & \text{if }v_{fs}<v_{b},\\ a\left[b{\left(v_{fs}-v_{b}\right)}^{3}-u_{fs}\right] & \text{if }v_{fs}\geq v_{b}, \end{array}$$

which enables the FSI model to exhibit Type 2 dynamics, such as a non-linear step at the start of the current-frequency curve between 0 and 15–20 spikes/s. Further discussion on the FSI model used in the striatal microcircuit can be found in Humphries et al. (2009b); the FSI model parameters are reproduced in Table 2.

**Table 2:** **Intrinsic parameters for the fast spiking interneuron model**.

**Parameter**	**Value**	**Source**
*a*	0.2	Izhikevich, 2007a
*b*	0.025	Izhikevich, 2007a
*d*	0	Izhikevich, 2007a
*k*	1	Izhikevich, 2007a
*v*_peak_	25 mV	Izhikevich, 2007a
*v*_*b*_	−55 mV	Izhikevich, 2007a
*C*	80 pF	Tateno et al., 2004
*c*	−60 mV	Tateno et al., 2004
*v*_*r*_	−70 mV	Tateno et al., 2004
*v*_*t*_	−50 mV	Tateno et al., 2004
η	0.1	Fitted to Bracci et al. (2002)
ϵ	0.625	Fitted to Gorelova et al. (2002)

*Dimensions are given where applicable. See Humphries et al. (2009b) for details*.

#### 2.1.3. Dopaminergic modulation of intrinsic ion channels

Tonic levels of dopamine in the striatum modulate the excitability of the projection neurons and fast-spiking interneurons (Nicola et al., 2000; Mallet et al., 2006). Our network model incorporates modulation by tonic dopamine through the relative activation levels of D1 and D2 receptors. These levels are modeled using the method proposed in Humphries et al. (2009b), in which complex membrane dynamics are subsumed by linear transforms with only two parameters ϕ_1_, ϕ_2_ ∈ [0, 1], describing the proportion of D1 and D2 receptor activation, respectively. Throughout we used ϕ_1_ = ϕ_2_ = 0.3.

For activation of D1 receptors on projection neurons we used the simple mappings:

(4) $$v_{r}\leftarrow v_{r}\left(1+K\phi_{1}\right)$$

and

(5) $$d\leftarrow d\left(1-L\phi_{1}\right),$$

which respectively model the D1-receptor mediated enhancement of the inward-rectifying potassium current(KIR) (Equation 4) and enhancement of the L-type Ca^2^+ current (Equation 5).

For activation of D2 receptors on projection neurons we used the mapping:

(6) $$k\leftarrow k\left(1-\alpha \phi_{2}\right)$$

which models the small inhibitory effect on the slow A-type potassium current, increasing the neuron's rheobase current (Moyer et al., 2007).

With these mappings, the model neuron is able to accurately capture the effect of D1 or D2 receptor activation on both the f-I curves and spiking input–output functions of the Moyer et al. (2007) compartmental model of the projection neuron.

Dopamine modulated fast spiking inter-neurons in the striatal network only express the D1-family of receptors (Centonze et al., 2003). Activation of this receptor depolarizes the neuron's resting potential [see Humphries et al. (2009b) for further details]. Thus we used the following mapping of the resting potential:

(7) $$v_{r}\leftarrow v_{r}\left(1-\eta \phi_{1}\right)$$

### 2.2. Synaptic models

Synaptic input comprises the source of current *I* in Equation (1):

(8) $$I=I_{\text{ampa}}+I_{\text{gaba}}+B(v)I_{\text{nmda}}.$$

where *I*_ampa_, *I*_gaba_, *I*_nmda_ are current input from AMPA, GABA, and NMDA receptors, respectively, and *B*(*v*) is a term that models the voltage-dependent magnesium plug in the NMDA receptors. Compared to the projection neuron, FSIs receive no NMDA receptor input from cortex, have a moderately larger AMPA conductance (Table 2), but do receive input via local gap junctions (see below).

Each synaptic input type *z* (where *z* is one of ampa, nmda, gaba) is modeled by

(9) $$I_{z}={\bar{g}}_{z}h_{z}\left(E_{z}-v\right),$$

where *g*_*z*_ is the maximum conductance and *E*_*z*_ is the reversal potential. We use the standard single-exponential model of post-synaptic currents

(10) $${\dot{h}}_{z}=\frac{-h_{z}}{\tau_{z}},\text{ and }h_{z}(t)\leftarrow h_{z}(t)+S_{z}(t),$$

where τ_*z*_ is the appropriate synaptic time constant, and *S*_*z*_(*t*) is the number of pre-synaptic spikes arriving at all the neuron's receptors of type *z* at time *t*.

Given that one interest here is in the possible roles of striatal NMDA sensitivity in Huntington's disease, we paid careful attention to two complexities of the NMDA receptor: its non-linear voltage-gating, and its saturation. The term *B*(*v*) in Equation (8), which models the voltage-dependent magnesium plug in the NMDA receptors, is given by (Jahr and Stevens, 1990)

(11) $$B(v)=\frac{1}{1+\frac{{\left[{\text{Mg}}^{2+}\right]}_{0}}{3.57}\text{exp}\left(-0.062v\right)},$$

where [Mg^2+^]_0_ is the equilibrium concentration of magnesium ions.

As glutamate can remain locked into the NMDA receptor for 100 ms or more (Lester et al., 1990), so the pool of available receptors becomes rapidly saturated at high afferent firing rates. To capture this we introduce a mean-field model of synaptic saturation where we interpret the term *h*_*z*_ in Equation (10) as the number of active receptor groups over the whole neuron. Each step in *h*_nmda_, following a number of spikes *S*_nmda_(*t*), activates that number of receptor groups, which decays with a time constant τ_nmda_. To introduce saturation, we bound the size of the step by the proportion of available groups. Together, these concepts give us the model:

(12) $${\dot{h}}_{\text{nmda}}=\frac{-h_{\text{nmda}}}{\tau_{\text{nmda}}},\text{ and }h_{\text{nmda}}(t)\leftarrow h_{\text{nmda}}(t)+\left[1-\frac{h_{\text{nmda}}(t)}{N_{\text{nmda}}}\right]S_{\text{nmda}}(t).$$

As well as introducing this saturation of the NMDA synapses, we also removed the 1/τ_*s*_ scaling of post-synaptic current amplitude used in Humphries et al. (2009a). This allowed the model synaptic conductances to be the same order of magnitude as their experimental counterparts. Consequently, we re-tuned *g*_ampa_ by fitting the input–output functions of the Moyer et al. (2007) 189-compartment projection neuron model, following the protocol in Humphries et al. (2009a). We obtained equally good fits to those found previously with a value of *g*_ampa_ = 0.4 (results not shown).

#### 2.2.1. Dopaminergic modulation of synaptic input

Following the projection neuron models in Humphries et al. (2009a), we add D1 receptor modulation of NMDA receptor evoked EPSPs by

(13) $$I_{\text{nmda}}^{\text{D1}}=I_{\text{nmda}}\left(1+\beta_{1}\phi_{1}\right),$$

and we add D2 receptor modulation of AMPA receptor evoked EPSPs by

(14) $$I_{\text{ampa}}^{\text{D2}}=I_{\text{ampa}}\left(1-\beta_{2}\phi_{2}\right),$$

where β_1_ and β_2_ are scaling coefficients determining the relationship between dopamine receptor occupancy and the effect magnitude (Table 3). Due to the addition of saturating NMDA synapses, we also re-tuned these parameter values by fitting the input–output functions of the Moyer et al. (2007) 189-compartment projection neuron model under D1 and D2 receptor modulation of synaptic inputs, following the protocol in Humphries et al. (2009a).

**Table 3:** **Synaptic and gap junction parameters for the striatal network**.

**Parameter**	**Value**	**Source and notes**
*E*_ampa_,*E*_nmda_	0 mV	Moyer et al., 2007
*E*_gaba_	−60 mV	Moyer et al., 2007
τ_ampa_	6 ms	Moyer et al., 2007
τ_nmda_	160 ms	Moyer et al., 2007
τ_gaba_	4 ms	Moyer et al., 2007
τ FSI gap	5	Fitted to Galarreta and Hestrin (1999)
[Mg^2+^]_0_	1 mM	Jahr and Stevens, 1990
*g*_ampa_ Ctx-SPN	0.4 nS	Tuning (see main text)
*g*_ampa_ Ctx-FSI	1 nS	Fits linear rise in EPSC data from Gittis et al. (2010)
*g*_nmda_ Ctx-SPN	0.2 nS	Fixed by maintaining the 2:1 AMPA:NMDA ratio from Moyer et al. (2007)
*g*_gaba_ SPN-SPN	0.75 nS	Koos et al., 2004
*g*_gaba_ FSI-SPN	3.75 nS	Mean 5-fold increase compared to SPN-SPN (Koos et al., 2004); 3× increase of PSP (Planert et al., 2010)
*g*_gaba_ FSI-FSI	1.1 nS	Gittis et al., 2010
*g* FSI gap	5 nS	Fitted to Galarreta and Hestrin (1999)
β_1_	0.5	Tuning (see main text)
β_2_	0.3	Tuning (see main text)

Finally, following the model in Humphries et al. (2009b), we add D2 receptor modulation of GABAergic input to FSIs by

(15) $$I_{\text{gaba}}^{\text{fsi}}=I_{\text{gaba}}\left(1-\epsilon_{2}\phi_{2}\right).$$

#### 2.2.2. Gap junctions

A gap junction between FSIs *i* and *j* is modeled as a compartment with voltage v^*^_ij_, which has dynamics

(16) $$\tau {\dot{v}}_{ij}^{*}=\left(v_{i}-v_{ij}^{*}\right)+\left(v_{j}-v_{ij}^{*}\right),$$

where τ is a time constant for voltage decay, and *v*_*i*_ and *v*_*j*_ are the membrane potentials of the FSI pair. The current introduced by that cable to the FSI pair is then

(17) $$I_{\text{gap}}^{*}(i)=g\left(v_{ij}^{*}-v_{i}\right)\;I_{\text{gap}}^{*}(j)=g\left(v_{ij}^{*}-v_{j}\right),$$

where *g* is the effective conductance of the gap junction. The total gap junction input *I*_gap_ to a FSI is then the sum over all contributions I^*^_gap_.

### 2.3. Striatum network model

**Figure 1:**
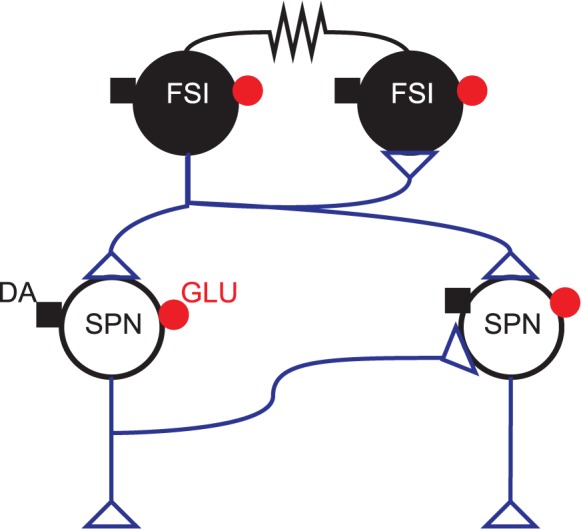
**GABAergic striatal microcircuit**. Input to the striatum comes from glutamatergic (GLU: •) fibers originating in the cortex, thalamus, hippocampal formation and amygdala, and dopaminergic (DA: ■) fibers from brainstem dopaminergic neurons. The projection neurons (SPNs) are interconnected via local collaterals of their axons projecting to other nuclei of the basal ganglia. The fast-spiking interneurons (FSIs) can form dendro-dendritic gap junctions between them and are also connected by standard axo-dendritic synapses. All these intra-striatal axo-dendritic connections (△) are GABAergic and hence inhibitory.

Our model captures the connections within the GABAergic microcircuit in striatum, illustrated in Figure 1. We simulated a large-scale model representing a three-dimensional cuboid of the striatum in the adult rat at one-to-one scale, containing every projection neuron and fast-spiking interneuron present in the biological tissue. We used a density of 89,000 projection neurons per mm^3^ (Oorschot, 1996) and a FSI density of 1% [see Humphries et al. (2010) for discussion]. We assumed projection neurons were evenly split between D1 and D2 receptor dominant types, and without any spatial bias. Hence we randomly assigned half of the projection neurons to be D1-type and half to be D2-type.

In the Results we predominantly report the results of simulations using a 300 μm on the side cube, giving 2292 projection neurons and 23 FSIs. Other sizes are noted explicitly where used.

To connect the neurons we used two different models. In the *physical* model we used distance-dependent functions for probability of connection between each element of the microcircuit. These functions were derived from overlap of dendritic and axonal arbors, and are given in Humphries et al. (2010) for each connection type in the microcircuit.

In the *random* model we ignored distance, and simply made connections to each neuron at random until the correct number of incoming connections of each type was made. The target number of connections were derived from the mean values obtained from the central neurons of the three-dimensional connectivity model in Humphries et al. (2010), and taken from column 1 of Table 5 in that paper: SPNs → 1 SPN: 728; FSIs → 1 SPN: 30.6; FSIs → 1 FSI: 12.8; FSI gap junctions per FSI: 0.65.

### 2.4. Selection competitions

Cortical input to the model was designed to emulate the response selection component in a general two-choice task, where a (possibly noisy) stimulus taking one of two values is observed over time and a choice made between the two corresponding responses. In such a task, we propose that the two responses are made salient by the onset of each trial and then, after a perceptual decision is made about the stimulus value, the corresponding response increases in salience. This generic setup was inspired by the experimental procedures of Beste et al. (2008), in which participants were asked to distinguish between short (200 ms) and long (400 ms) auditory tones, using a distraction paradigm. Inputs followed a ramping trajectory to simulate evidence accumulation and increasing decision confidence (Asaad et al., 2000). We previously showed that transient selection can be seen in response to stepped cortical inputs (Tomkins et al., 2012).

The striatum model was divided up into three populations, two physically close SPN populations representing the two competing responses, which we refer to throughout as *channels*, and the remaining background neurons given a constant input. Neurons were randomly divided into the two channels, with 40% of the neurons in channel 1 and 2, respectively, and the remaining 20% of cells were labeled “background” neurons.

**Figure 2:**
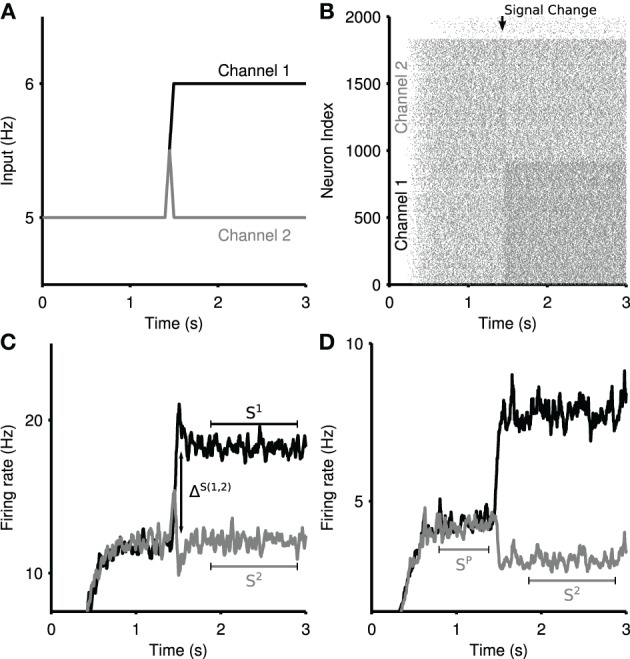
**Measures of selectivity in striatal output**. **(A)** Ramping cortical input into the striatum model. Two channels are driven by input spike trains, demonstrating signal selection between most-salient (channel 1) and least-salient (channel 2) striatal signals. **(B)** Raster plot of the striatum microcircuit output for a single selection experiment. Increased firing can be seen in channel 1 at the onset of the ramped input in panel **(A)**. **(C)** A sample striatal output of the *physical* network, showing a zero-phase filter of the mean spiking output from the two competing channels in response to the ramped input in panel **(A)**. Annotations demonstrate the measures used in the transient selectivity measure. *S*^1^, *S*^2^: stable firing rate; Δ^*S*(1,2)^: maximum of the difference between the two channels firing rates over the transient period. **(D)** A sample striatal output of the *random* network, in response to the same input. Annotations demonstrate the measures used in the steady-state selectivity measure. *S*^*P*^: pre-step stable firing rate.

The input protocol is illustrated in Figure 2A, and Figure 2B shows an example response of the entire network to this protocol. Each response population received a priming input at a background rate for 1500 ms, causing them to reach a steady-state of firing activity. At 1500 ms, channel 1, (black) received a ramping input for a time of 50 ms, raising the salience toward a new steady-state, when it became the most salient cortical input to the striatum. During the 50 ms ramping time, channel 2 also received a ramping input, matching that of channel 1 for 25 ms. Following this, the signal to channel 2 decreased back to the background rate, describing the evidence accumulation trajectory of an out-competed action.

Rates were specified for each cortical spike train input to each projection neuron and FSI model. Both neuron models received the equivalent of 250 input spike trains [see Humphries et al. (2009b) for details].

We measured how the striatal microcircuit performed channel wise signal selection on the cortical inputs, using this simple protocol, inspired by the auditory decision task performed in Beste et al. (2008). However, due to the abstract nature of the input protocol we use, applied to a generic simulation of the striatal microcircuit, the selection measured in these results could be applied to any channel-wise decision task throughout the striatum, and is not limited to auditory processing.

### 2.5. Metrics for selection

We define “selectivity” in the striatum as the ability to robustly distinguish competing signals. The striatum demonstrates two complementary modes of selectivity, which we measure with different metrics. These selection metrics are applied to the output of each channel, which is characterized by a zero-phase filtered mean firing rate.

#### 2.5.1. Transient selectivity

Given a competitive split in cortical input, we see a temporary boosting of the most-salient signal, accompanied with a temporary suppression of the least-salient competitive signal (Figure 2C). This transient phenomena presents a boost of the difference in salience between the two competing signals. We identify two key regimes: (1) Δ^*S*(1,2)^, the maximum difference between the two signals during the transient peaks; (2) *S*^1^, *S*^2^, the mean stable activity level of each channel after the transient period dissipates. The total transient selectivity, between 0 and 1, is defined as

(18) $$TS=1-\frac{S^{1}-S^{2}}{\Delta^{S(1,2)}},\;0\leq TS\leq 1$$

where Δ^*S*(1,2)^ is the *maximum* difference between the firing rates of Channel 1 and Channel 2 over the transient window (*t* = 1500 : 2000 ms). This enables the measure to allow for cases in which the largest perturbations from the mean are not temporally coincident, either due to reliable intrinsic dynamic properties of the network, or statistical fluctuations therein.

#### 2.5.2. Steady-state selectivity

The striatum network can exhibit signal suppression on its least-salient channel due to sustained inhibition by the most salient channel. *Steady-state* selectivity is measured on the least-salient channel, as the percentage reduction in the mean channel firing rate after the rise in salience of the most-salient signal. An example of steady-state selectivity in the *random* network can be seen in Figure 2D. We define (*S*^*P*^) as the stable firing rate of the primed channel 2 before the increase in competition, and from this we calculate the steady-state selectivity (SS) as:

(19) $$SS=100\times \left(1-\frac{S^{2}}{S^{P}}\right).$$

### 2.6. Basal ganglia-thalamocortical loop model of transient selection

**Figure 3:**
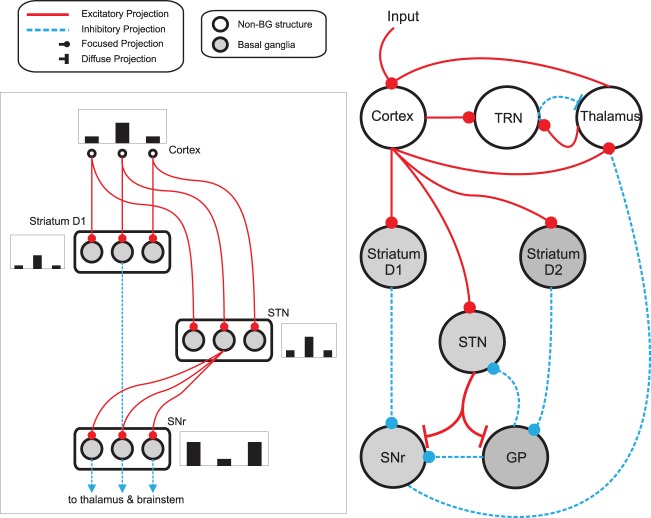
**Basal ganglia thalamo-cortical loop model**. The main circuit (right) embeds the basal ganglia into a thalamo-cortical feedback loop. Each nucleus contains multiple response-representing populations. Within the basal ganglia, the circuit can decomposed into an off-center, on-surround network (left): three populations are shown, with example activity levels in the bar charts to illustrate the relative contributions of the nuclei. Note that, for clarity, full connectivity is only shown for the second population. Briefly, the selection mechanism works as follows. Constant inhibitory output from substantia nigra pars reticulata (SNr) provides an “off” signal to its widespread targets in the thalamus and brainstem. Cortical inputs representing competing saliences are organized in separate populations, which project to corresponding populations in striatum and subthalamic nucleus (STN). The balance of focussed inhibition from striatum and diffuse excitation from STN results in the most salient input suppressing the inhibitory output from the corresponding SNr population, signaling “on” to that SNr population's targets. Tonic dopamine levels in the striatum set the ease with which the channels are selected, and subsequently switched between following further salient inputs. For quantitative demonstrations of this model see Gurney et al. (2001b) and Humphries and Gurney (2002). GP: globus pallidus; SNr: substantia nigra pars reticulata; STN: subthalamic nucleus; TRN: thalamic reticular nucleus.

To study the contribution of the transient striatal dynamics to the selection mechanism of the whole basal ganglia, we used the population-level implementation of our basal-ganglia thalamo-cortical loop model (Humphries and Gurney, 2002). Figure 3 schematically illustrates the loop model, and the connectivity of the response-representing populations.

The average activity *a* of all neurons comprising a channel's population changes according to

(20) $$\tau \dot{a}=-a(t)+I(t)$$

where τ is a time constant and *I* is summed, weighted input. We used τ = 10 ms throughout. The normalized firing rate *y* of the unit is given by a piecewise linear output function

(21) $$y(t)=F\left(a(t),\;\theta \right)=\{\begin{array}{ll} 0 & a(t)\leq \theta \\ a(t)-\theta & \theta <a(t)<1-\theta \\ 1 & a(t)\geq 1-\theta \end{array}$$

with threshold θ.

The following describes net input *I*_*i*_ and output *y*_*i*_ for the *i*th channel of each structure, with *n* channels in total. The full model was thus given by (Humphries and Gurney, 2002):

$$\begin{array}{l} \text{ Cortex: }I_{i}^{\text{ctx}}=y_{i}^{\text{thal}}+c_{i},\\ \;y_{i}^{\text{ctx}}=F\left(a_{i}^{\text{ctx}},\;0\right),\\ \text{ Thalamus: }I_{i}^{\text{thal}}=y_{i}^{\text{ctx}}-y_{i}^{\text{SNr}}-0.1y_{i}^{\text{TRN}}\\ \;-0.7\sum_{j\neq i}^{n}y_{j}^{\text{TRN}},\\ \;y_{i}^{\text{ctx}}=F\left(a_{i}^{\text{thal}},\;0\right),\\ \text{ TRN: }I_{i}^{\text{TRN}}=y_{i}^{\text{thal}}+y_{i}^{\text{ctx}},\\ \;y_{i}^{\text{TRN}}=F\left(a_{i}^{\text{TRN}},\;0\right),\\ \text{ Striatum D1: }I_{i}^{d1}=y_{i}^{\text{ctx}}\left(1+\lambda_{1}\right),\\ \;y_{i}^{d1}=F(a_{i}^{d1},\;0.2),\\ \text{ Striatum D2: }I_{i}^{d2}=y_{i}^{\text{ctx}}\left(1-\lambda_{2}\right),\\ \;y_{i}^{d2}=F\left(a_{i}^{d2},\;0.2\right),\\ \text{Subthalamic nucleus: }I_{i}^{\text{stn}}=y_{i}^{\text{ctx}}-y_{i}^{\text{gp}},\\ \;y_{i}^{\text{stn}}=F\left(a_{i}^{\text{stn}},\;-0.25\right),\\ \text{ Globus pallidus: }I_{i}^{\text{gp}}=0.9\sum_{j}^{n}y_{j}^{\text{stn}}-y_{i}^{d2}\\ \;y_{i}^{\text{gp}}=F\left(a_{i}^{\text{gp}},\;-0.2\right),\\ \text{ SNr: }I_{i}^{\text{snr}}=0.9\sum_{j}^{n}y_{j}^{\text{stn}}-y_{i}^{d1}-0.3y_{i}^{\text{gp}},\\ \;y_{i}^{\text{snr}}=F\left(a_{i}^{\text{snr}},\;-0.2\right), \end{array}$$

Net input was computed from the outputs of the other structures, except driving input *c*_*i*_ to channel *i* of cortex. The striatum was divided into two populations, one of projection neurons with the D1-type dopamine receptor, and one of projection neurons with the D2-type dopamine receptor. Many converging lines of evidence from electrophysiological and anatomical studies support this functional split into D1- and D2-dominant projection neurons and, further, that the D1-dominant neurons project to SNr, and the D2- dominant neurons project to GP (Gerfen et al., 1990; Surmeier et al., 2007; Matamales et al., 2009).

In line with the projection neuron model described above, the model included opposite effects of activating D1 and D2 receptors on striatal projection neuron activity: D1 activation facilitated cortical efficacy at the input, while D2 activation attenuated this efficacy (Moyer et al., 2007; Humphries et al., 2009a). The mechanism for this mirrored that of the spiking projection neuron model in using simple linear factors. Thus, if the relative activation of D1 and D2 receptors by tonic dopamine are λ_1_, λ_2_ ∈ [0, 1], then the increase in efficacy due to D1 receptor activation was given by (1 + λ_1_); the decrease in efficacy due to D2 receptor activation was given by (1 − λ_2_). Throughout we set λ_1_ = λ_2_ = 0.2, simulating tonic levels of dopamine.

The negative thresholds ensured that STN, GP, and SNr have spontaneous tonic output (Humphries et al., 2006). We simplified the model here compared to Humphries and Gurney (2002) by delivering input only to cortex, to represent the salience-driven response selection, rather than to cortex, striatum and STN; both models gave qualitatively the same results. We used exponential Euler to numerically solve this system, with a time-step of 1 ms.

We used *n* = 8 channels in total, with two of those channels (4 and 5) receiving non-zero inputs, mimicking the input protocol used for the striatal network model, which is designed to abstractly simulate the two choice reaction-time task performed in Beste et al. (2008). Baseline inputs *c*_4_ = c_5_ = 0.3 were delivered at simulation onset. A step in input *c*_5_ occurred between 100 and 200 time-steps: a small step of *c*_5_ = 0.5 or a large step of *c*_5_ = 0.7. The ability for the model to select was assessed during this step period. As in prior models (Berns and Sejnowski, 1998; Gurney et al., 2001b; Humphries and Gurney, 2002; Humphries et al., 2006), selection was assessed by observing the change in activity on each SNr channel, as this output provides the tonic inhibition of thalamic and brainstem structures and is thought to gate the execution of actions (Redgrave et al., 1999). Here, successful selection of a channel was defined as the SNr output falling to zero.

#### 2.6.1. Modeling transient selection in the rate-coded model

We mimicked the ability of the striatum microcircuit to produce transient phenomena using an input injection into the striatum of the rate coded model. At *t* = 100 we injected external inputs into each striatal channel in the model, forcing a transient increase or decrease as appropriate in the corresponding channels. Transient sizes were extracted from the striatal microcircuit traces, and reproduced in the rate coded model. Individual transients were calculated as the percentage change in the firing rate of the circuit during the transient period compared to the stable firing rate achieved post-transient. This allowed us to gauge the role of the complex striatal dynamics, generated by our microcircuit model and responsible for the transient selection mechanism, on the selection properties of the entire basal ganglia-cortex loop.

## 3. Results

In what follows we discuss the simulation results of our model and interpret them as potential mechanisms explaining the findings of Beste et al. (2008). We discuss the two types of potential selection mechanisms that we have termed *transient* and *steady-state*.

### 3.1. Transient selection by the striatum

#### 3.1.1. Transient selection emerges from the striatal microcircuit

We sought insight into the potential for competition within the striatum by examining the dynamics of our three-dimensional network model. We first explored the effect on striatal output of competing inputs to two projection neuron populations. These inputs were intended to emulate the changes in cortical signals representing two alternative responses in a generic two-choice decision-making task.

**Figure 4:**
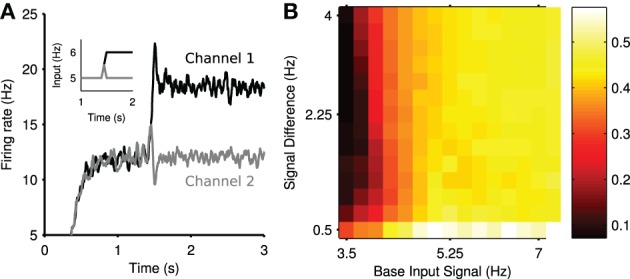
**Transient selection of competing input signals by the striatum**. **(A)** Mean firing rate of the two output channel populations in the experiment in response to the ramped input protocol (inset); individual spike trains have been convolved with a zero-phase digital filter to create smooth firing rates without lag. **(B)** Mean transient selection landscape color coded such that brighter colors represent higher selectivity. Landscape shows the mean transient selectivity averaged over 30 trials as a function of base input signal and step in signal difference during competition.

Figure 4A shows the mean firing rate of each channel from the same example simulation. After the divergence in inputs at *t* = 1.5 s, a transient increase of the firing rate is elicited in channel 1, the most salient population, and a transient suppression of the firing rate is elicited in channel 2. This transient suppression occurs despite no change in the input to channel 2. Moreover, this population rapidly returns (~100 ms) to its pre-step firing rate. Consequently, we termed this phenomenon *transient* selection.

We found that the elicited transient selection was robust over a wide range of choices for the baseline input rate and the signal difference between the two channel inputs after the signal divergence. Figure 4B shows that transient selection could be robustly elicited for any step size over 0.5 Hz when the baseline input rate exceeded ~4 Hz.

#### 3.1.2. Transient selection is due to both circuit and intrinsic membrane properties

**Figure 5:**
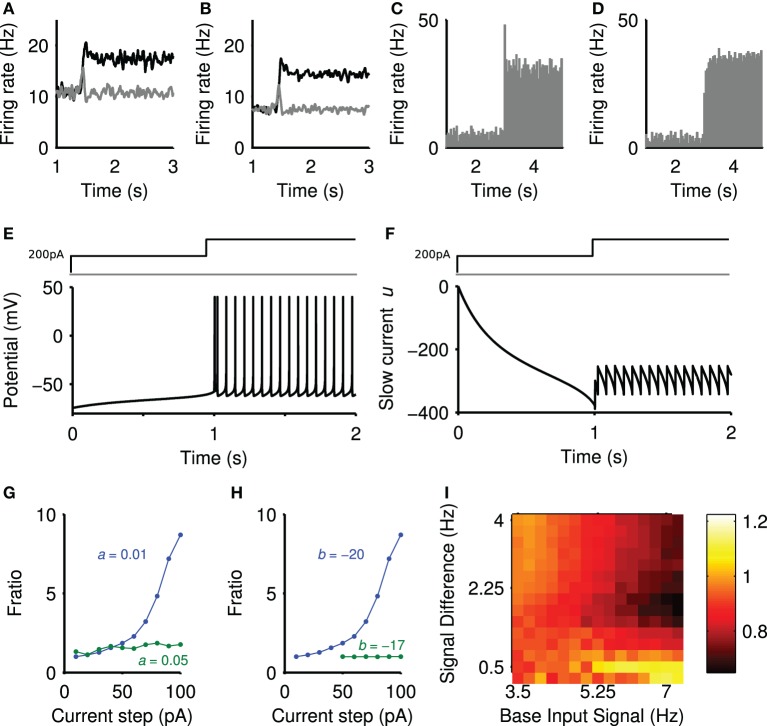
**Sources of the positive and negative transients**. **(A)** Striatal output with lesioned projection neuron connections. **(B)** Striatal output with all intra-striatal connections lesioned. **(C)** Peri-stimulus time histogram of a single projection neuron output, averaged over 50 steps of spiking input from *r* = 4 Hz to *r* = 7.2 Hz (onset *t* = 3 s), exhibiting transient behavior. **(D)** Peri-stimulus time histogram of a single regular-spiking cortical neuron model, averaged over 50 steps of spiking input from *r* = 0.75 Hz to *r* = 3 Hz, with no transient behavior. Model parameters given in Izhikevich (2007a). **(E)** The membrane potential (*v*) of the projection neuron model in response to a depolarizing current injection (200 pA) followed by a further step in current at 1 s. **(F)** The corresponding changes in the slow current (*u*). **(G)** *f*_ratio_ in the projection neuron model as a function of current step size and slow current decay constant 1/a ms. **(H)** *f*_ratio_ in the projection neuron model as a function of current step size and slow current gain *b*. **(I)** The effect of projection neuron connection lesions on the negative transient. Landscape of negative transients measured as ratio of the maximum negative transient peak over the steady-state, plotted as a function of base input rate vs signal difference.

We further investigated the mechanisms underlying the positive and negative transient changes in population activity. We found that the positive transient was produced by single neuron dynamics, whereas the negative transient was due to network connectivity. This can be seen in Figures 5A,B, where lesioning either the projection neuron connections or all the network connections abolished the negative transient but did not prevent the positive transient.

To confirm the positive transient was a single neuron phenomenon, we simulated an individual projection neuron model receiving many trials of the same stepped input protocol, and averaged its responses. The resulting peri-stimulus time histogram (Figure 5C) shows that the neuron had a clear transient increase in firing probability immediately after the step of input. Running the same test on a model of a cortical regular-spiking pyramidal neuron, with input scaled to produce approximately the same steady-state rates, showed no such transient increase in firing probability after a step in input (Figure 5D). Thus the transient increase in population activity observed in a single trial of the network is a statistical phenomenon of synchronous spiking of many projection neurons, and seemingly dependent upon properties particular to the striatal projection neuron.

We sought to elucidate these properties by injecting sequential current steps directly into the projection neuron model and observing the behavior of the membrane voltage *v* and slow current *u*. Figure 5E shows that a step in current applied to an already depolarized membrane triggers a rapid double spike, followed by slower regular spiking. Figure 5F plots the corresponding trajectory of the slow current *u*: the initial depolarizing injection makes the slow current *u* increasingly negative, thus slowly charging the membrane potential *v* [Figure 5E; see Equation (1)]. The subsequent step of injected current increases the membrane potential rapidly, and the contribution of the large, negative *u* ensures a rapid pair of spikes time-locked to the current step. However, once spiking has been initiated, the equilibrium value of *u* is less negative than immediately before the current step. Consequently, the smaller contribution of the slow current *u* ensures a comparatively slow spike rate in the steady-state.

To show that the slow current *u* is critical, we examined the dependence of this spiking “adaptation” on the parameters of the slow current. We repeated the sequential-step current injection protocol for a range of step-sizes, and measured the adapting response as *f*_ratio_ = *F*_first_/*F*_last_, the ratio of the first and last inter-spike intervals after the current step. A value of *f*_ratio_ > 1 thus indicates an adaptation. We found that the adaptation response appeared with a second current step above ~50 pA (blue curves in Figures 5G,H). Figure 5G shows that the adaptation response disappeared if we reduced the effective time constant of the slow current (increased *a*), allowing the slow current to recover faster after spiking. Figure 5H shows that the adaptation response also disappeared if we reduced the gain *b* of the slow current The transient phenomena thus depends critically on the slow current *u*.

As lesioning only the connections between the projection neuron could abolish the negative transient (Figure 5A), this suggested it arose from a network effect where the neurons contributing to the positive transient inhibited their targets. To test this observation, we simulated the model with lesioned projection-neuron collaterals for a range of baseline input firing rates and step sizes (protocol in Figure 2A) and computed the size of the negative transient that resulted. Figure 5I shows that the negative transient was indeed abolished for a wide-range of values for the input firing rates. However, a sufficiently large baseline firing rate and step in firing rate could still result in a negative transient (upper-right corner of Figure 5I). Thus, it seems that sufficient cortical drive of the FSI population (which inhibits the projection neurons) also contributes to the negative transient in projection neuron population activity.

#### 3.1.3. Transient selection is sufficient to alter decision making performance

Though the previous result demonstrates the existence and origin of transient selection within the striatum, it is not sufficient to show a causative effect of transient selection on decision-making. To address this issue, we asked whether such transient signals in the striatum could enhance the selection of input signals by the basal ganglia circuit. Here we consider selection to mean that the output of a substantia nigra pars reticulata (SNr) population falls from its tonic rate to zero. In particular, we hypothesized that the transient signals in striatum would be amplified in the complete basal-ganglia-thalamo-cortical loop, and thus directly influence the output of the basal ganglia.

**Figure 6:**
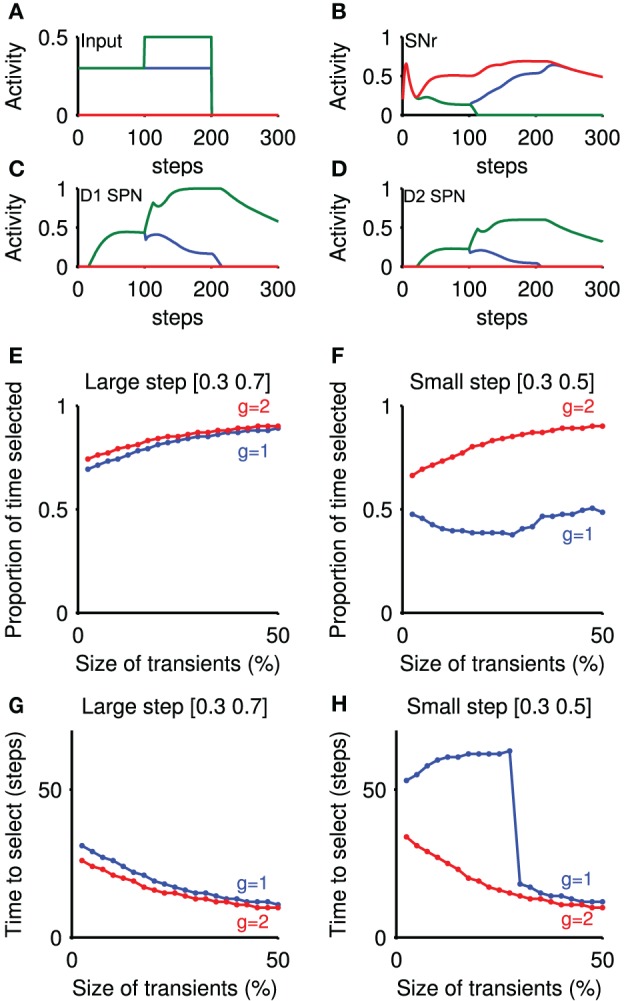
**Transient selection in striatum is amplified by basal ganglia-thalamo-cortical loop**. Panels **(A–D)** show an example simulation of the loop model that included emulation of the transient selection signals originating in the striatum (transient size: 50%; thalamo-cortical loop gain *g* = 2). **(A)** Cortical input to the rate-coded model, mimicking the selection protocol used in the striatal microcircuit selection experiments. **(B)** Corresponding SNr output response for three populations: no input (red); baseline only (blue); and baseline-plus-step (green). The input step thus caused clear selection by forcing the SNr output to zero. **(C)** Evoked response in the rate coded striatal D1 neurons, showing the effect of the injected transient at *t* = 100. **(D)** Evoked response in the rate coded striatal D2 neurons. **(E)** Proportion of time an action was selected, as a function of transient size. Transient size is expressed as a proportion of the steady-state firing rate achieved without the transient. Step values indicate the cortical input before and after the step in input. Parameter *g*: closed-loop gain of the thalamocortical loop. **(F)** Proportion of time an action was selected, given a small input step. **(G)** Time delay before selection achieved, as a function of transient size, for large input step. Delay is given between the step in cortical input and the corresponding SNr population reaching zero output. **(H)** Time delay before selection achieved, as a function of transient size, for small input step.

To test this, we used our rate-coded model of population activity in the basal ganglia-thalamocortical loop (Humphries and Gurney, 2002). The model received inputs to two populations of cortico-striatal neurons (Figure 6A), mimicking the protocol used in our full-scale striatum model. An example of the subsequent SNr outputs are illustrated in Figure 6B. At the time of the step in input to one population, we emulated the subsequent transient signals observed in our full-scale model by brief injections of further increased input to that striatal population and decreased input to the other. These correspondingly produced small, brief positive and negative transients in the output of those striatal populations, for both D1 and D2-type projection neurons (Figures 6C,D). Note that the subthalamic nucleus populations also received the cortical input signals, but not the transient signals.

We found that a small positive transient elicited in the striatal population was sufficient to change the speed and persistence of selection (Figures 6E–H). Figures 6E,F show that signal selection was maintained for longer with increasing transient sizes. Correspondingly, Figures 6G,H show that increasing the size of transients injected into the model striatum decreased the time to selection. These changes were found irrespective of the size of input step, or of the closed-loop gain *g* of the positive thalamocortical feedback loop (Chambers et al., 2011) (When *g* = 1, this loop is a perfect integrator, while with *g* = 2, there is an amplifying feedback loop.) Thus, transient signals in the striatum are sufficient to modulate selection by the basal ganglia.

### 3.2. Steady-state selection by the striatum

Prior debates about selection in the striatum have focussed on stable, winner-take-all modes of computation (Wickens, 1997; Plenz, 2003). In order to compare transient selection with this more common form of selection computations, we sought to understand whether our striatal model could show stable, winner-takes-all-like dynamics; here we refer to these as “steady-state” selection, in contrast to “transient” selection, as the competition between inputs causes persistent changes to output firing rates.

#### 3.2.1. Steady-state selection in a randomly-connected model

Neurally-inspired models of winner-take-all dynamics are often based on fully-connected or dense randomly-connected networks (Hartline and Ratliff, 1958; Alexander and Wickens, 1993; Fukai and Tanaka, 1997; Mao and Massaquoi, 2007; Yim et al., 2011). We thus simulated our striatal model with random connectivity, in which each neuron type received, on average, the same number of connections, and the connections were made by choosing source neurons at random from across the three-dimensional cuboid. The target number of connections was based on the expected number of connections of a projection neuron and FSI in the center of a 1 mm^3^ network, according to the computational anatomical estimates of Humphries et al. (2010) (see Materials and Methods). In this way, the randomly-connected model was more densely connected relative to the distance-dependent model. Thus, while closer to the topology usually studied for steady-state selection, the randomly-connected model still retained connection statistics consistent with the estimates obtained in Humphries et al. (2010).

**Figure 7:**
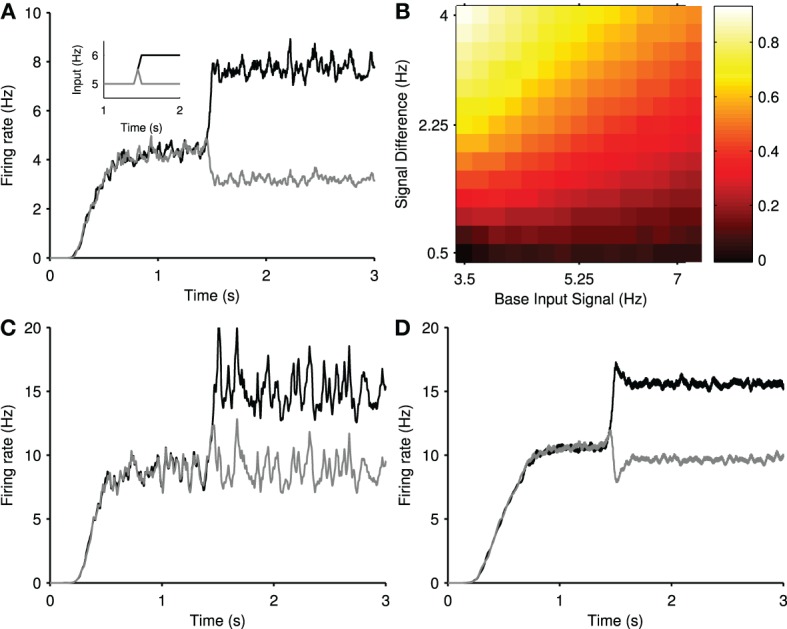
**Steady-state selection in the randomly-connected striatum model**. **(A)** Smoothed mean firing rates of two projection neuron populations, in response to the ramped input protocol (inset). **(B)** The magnitude of steady-state selection as a function of baseline input and step size. The magnitude gives the fall in firing rate of the losing population as a proportion of its pre-step firing rate. Each magnitude is an average over 15 simulations. **(C)** Smoothed mean firing rates of two projection neuron populations, with SPN-SPN connections lesioned, in response to the same input as above. Steady-state selectivity is removed. **(D)** Smoothed mean firing rates of two projection neuron populations, with FSI-SPN connections lesioned, in response to the same input as before. Steady-state selection remains.

We tested the randomly-connected model with the same stepped input protocol as the physically-connected model (Figure 2A). Figure 7A shows an example of the mean population firing rates in the randomly-connected striatum model, with evident steady-state selection: the population receiving the stepped cortical input increases its firing rate, and the other population correspondingly decreases its firing rate despite receiving the same input throughout. We found that the magnitude of steady-state selection was dependent on the size of the baseline firing rate and input step. Figure 7B shows that the most effective steady-state selection occurred for low baseline rates and large input steps, approaching a winner-takes-all like response of nearly complete suppression (~80%) of the losing population's activity.

Figure 7C shows that lesioning the connections between projection neurons prevents steady-state selection. Figure 7D shows that lesioning the FSI input to the projection neurons reduces but does not eliminate the steady-state selection, while also reinstating a transient period. This suggests that mutual inter-channel inhibition by the projection neurons populations is responsible for the suppression effect seen in both the *random* and the larger *physical* networks.

#### 3.2.2. Distance-dependent connectivity can support steady-state selection

**Figure 8:**
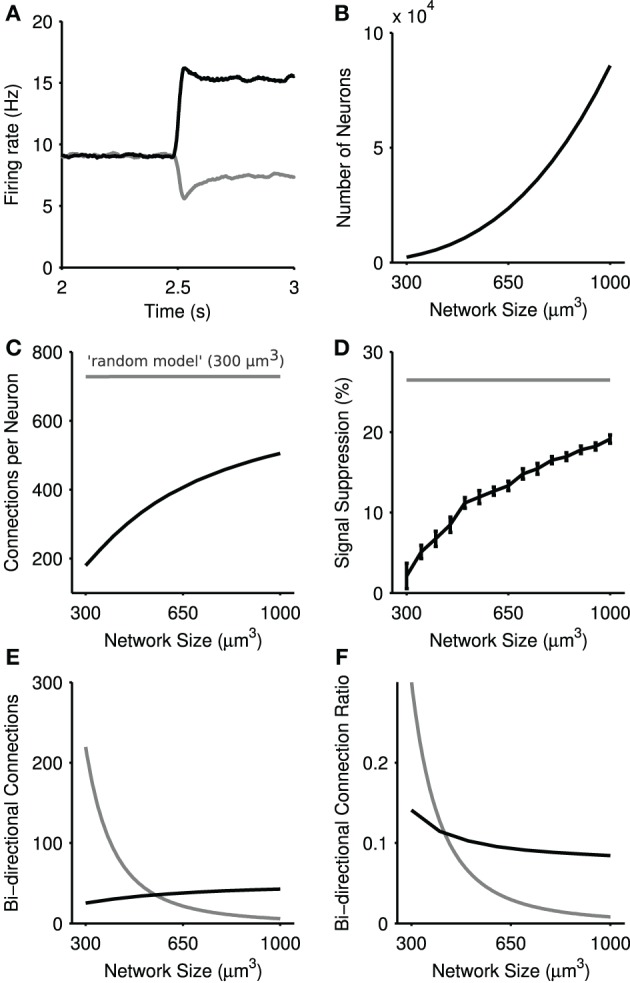
**Steady-state selection in the physical model of the striatal microcircuit**. **(A)** Mean firing rate of two projection neuron populations in a 1 mm^3^ model, with 89,749 total simulated neurons. **(B)** Number of simulated neurons as a function of network size. **(C)** Average number of connections per neuron as a function of network size. The *physical* network (black) approaches the density of connections seen in the random network (gray) with increased network size. **(D)** Magnitude of steady-state selection as a function of network size. All simulations used the inputs [5,6] Hz. Magnitude is the percentage suppression in the average firing rate of the losing channel after the competitive signal onset (*t* = 2.5 s). Shown in gray is the steady-state selectivity seen in the random model for a network of size 300 μm^3^. Bars set at ± 2 s.d, computed over 15 repeats. **(E)** Number of bi-directional connections as a function of network size. The total number of pairs of reciprocal connections in the *physical* model are shown in black, and the *random* model in gray. Bi-directional pairs decrease in the physical model with increasing network sizes, due to the fixed number of connections each neuron receives. **(F)** The ratio *R*_*bi*_ of bi-directional connections to the total number of connections a neuron makes for the *physical* model (black) and the *random* model (gray).

To assess if such steady-state selection required homogeneous, random connectivity of the kind described above, we checked whether such selection could be found in the physical model of connectivity. Again using the same stepped input protocol, we simulated physical networks up to 1 mm^3^, in order to increase the density of connectivity within the center of the network, which scales with the number of neurons in the model (Figure 8B).

Figure 8A shows that steady-state selection could be observed for distance-dependent connectivity, given a sufficiently large model (here 1 mm^3^). We found that the magnitude of steady-state selection increased monotonically with increasing network size (Figure 8D), approaching the steady-state selectivity seen in the *random model*. Figures 8B,C shows that in the *physical* model as the number of neurons increases as a function of network size so does the average number of connections each projection neuron receives. By contrast, the *random model* always has the same density of connections. The *physical* model's correspondence between the number of connections to a projection neuron and the effectiveness of steady-state selection suggests that such selection is dependent on the density of connections between projection neurons.

The model further suggests that it is only the increased density of connections that is key, and not an increase in recurrent connections between projection neurons. Figure 8E shows the absolute number of recurrent connections in the *physical* and *random* network configurations. Note that the number of bi-directional connections in the *random* network drops of as a function of network size due to the fact that each neuron receives a fixed number of connections regardless of the network size. By contrast we see a small rise in the number of bi-directional connections in the *physical* model. However, Figure 8F shows that in both random and physical networks the proportion of connections that are bi-directional falls with increasing network size. Thus, the increased effectiveness of steady-state selection is likely due to increased absolute connection density and not increased recurrent connections.

### 3.3. Comparing selection mechanisms: paradoxical selection enhancement in huntington's disease

Having established that two contrasting forms of selection can be supported by the striatal circuit, depending on the type and density of connectivity, we then sought insight into how the two forms of selection could be distinguished. In particular, we hypothesized that they would make different predictions about how changes to the striatum would alter response selection. In order to test this hypothesis, we sought an experimental data-set that could provide a basis for testing our predictions.

Beste et al. (2008) have recently shown a rare example of paradoxical cognitive enhancement in a neurological disorder. They reported that manifest Huntington's disease patients had faster and less error prone response selection on a simple two-choice auditory task than controls or pre-manifest Huntington's disease patients. As Huntington's disease is primarily characterized by widespread loss of striatal projection neurons [FSI populations have been shown to be more resistant to HD-modifications (Ghiglieri et al., 2012)], and increased sensitivity of NMDA receptors on striatal projection neurons (Fan and Raymond, 2007), these results suggest the hypothesis that one or both of these changes to the striatum lead to enhanced selection, and as such we look into excitotoxicity as a possible candidate for the paradoxical improvements investigated.

We thus simulated both transient and steady-state selection under Huntington's-like changes to the striatal model, and searched for evidence of enhanced selection. We emulated increased NMDA receptor sensitivity by increasing the conductance of the NMDA synapse (we report this as the ratio of the NMDA:AMPA conductances), and separately emulated the cell loss by randomly removing a specified percentage of projection neurons. We did this to explore a wide range of plausible simulated Huntington's disease conditions. Across both changes, we mapped the change in transient and steady-state selection in response to the same input protocol (baseline 5 Hz, step 1 Hz).

#### 3.3.1. Steady-state selection consistently degrades in simulated huntington's disease

**Figure 9:**
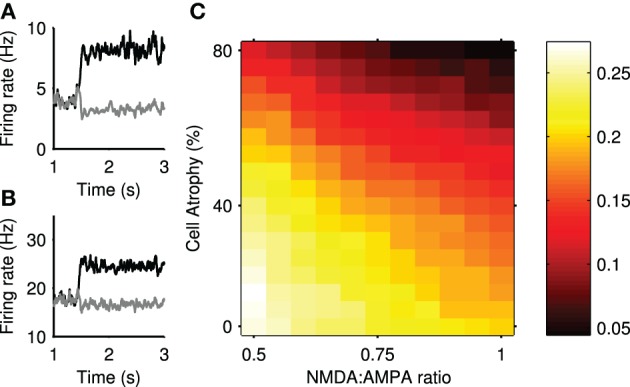
**Steady-state selection under simulated Huntington's disease**. **(A)** An example of reduced signal suppression in the striatum with high cell atrophy (65% cell loss, NMDA:AMPA ratio 0.5). **(B)** An example of removed signal suppression in the striatum with high degradation (75% cell loss, NMDA:AMPA ratio 1). **(C)** Magnitude of signal suppression over all simulated Huntington's conditions. Magnitudes are means over 15 simulations. The control, healthy-state model is in the bottom left-hand corner (NMDA:AMPA = 0.5; 0% atrophy).

To assess the impact of Huntington's-like changes on steady-state selection, we used the randomly-connected model to ensure that the suppression of the losing population was sufficient to be detectably modulated by the Huntington's-like changes. Figure 9 shows that steady-state selection was uniformly diminished by all Huntington's-like changes, whether in isolation or combination.

#### 3.3.2. Transient selection enhancement in simulated huntington's disease

**Figure 10:**
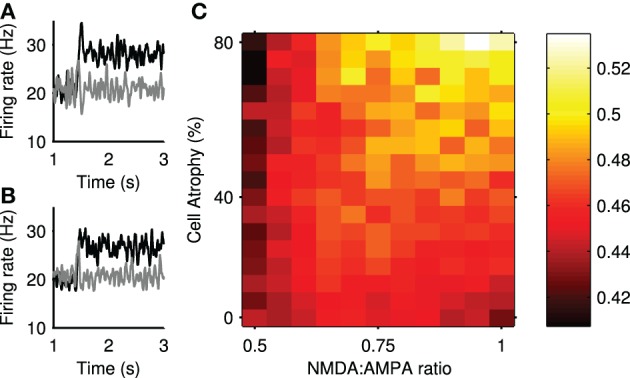
**Transient selection can be enhanced in simulated Huntington's disease**. **(A)** An example of enhanced transient selection in a Huntington's-like condition (81% cell atrophy, 0.95 NMDA:AMPA ratio) **(B)** An example of the loss of transient selection in a Huntington's-like condition (81% cell atrophy, 0.55 NMDA:AMPA ratio). **(C)** Selection landscape for NMDA:AMPA conductance ratio against cell atrophy. Color coded such that brighter colors represent better transient selectivity in the striatal model. Magnitudes are means over 30 simulations. The control, healthy-state model is in the bottom left-hand corner (NMDA:AMPA = 0.5; 0% atrophy).

We assessed the impact of Huntington's-like changes on transient selection using the same physical model network as that used for Figure 4. Figure 10 shows that transient selection could be diminished by the loss of projection neurons alone, yet could be enhanced by the simultaneous increase in NMDA conductance. Thus the model predicts a region of Huntington's-like conditions where the deleterious effect of cell loss can be more than compensated by the increased sensitivity of NMDA receptors.

Figure 10A shows an example improvement in transient selectivity under high cell atrophy and a high excitability, whereas Figure 10B shows the removal of the transient selectivity under high cell atrophy but only a small increase in excitability. These examples show that the transient selectivity range of ~0.10 over the “excitotoxicity landscape” in Figure 10C, corresponds to dramatic changes in the striatal output. Further, Figure 6 shows that even small modifications in the transient size in the striatum will modulate the signal selection speed in the wider basal ganglia networks.

## 4. Discussion

We found a novel form of transient selection supported by the striatal network. This emerged from our three-dimensional network of sparse, weak feedback connectivity between the striatal projection neurons and dense, strong feedforward inputs from the fast-spiking interneurons. We observed that rapidly increasing the ongoing input to one of two competing populations of projection neurons caused a transient peak of activity in that population and a synchronous transient dip in activity of the other. The dip lasted around 100 ms before the activity returned to its pre-step level, thus showing no steady-state competitive effect between the two populations.

Using a population-level model of the complete basal ganglia-thalamo-cortical loop, we showed that transient selection in the striatum was sufficient to enhance selection by the entire circuit (as determined by suppression of SNr output). The presence of transient selection both increased the speed at which the whole circuit resolved a competition between salient inputs, and increased the circuit's ability to persist with the selected input. Both effects were observed for either perfect-integrator or amplifying feedback in the thalamo-cortical loop.

The origin of the transient selection had two components. The positive transient in the population activity was driven by single neuron adaptation. We found that a further step in input to an already depolarized projection neuron caused a spike followed by rapid decrease in spiking probability. This implies that the positive transient observed in the population activity was a statistical effect: that, across a whole population of projection neurons, a sub-set of neurons were sufficiently depolarized at the time of stepped input to show this adaptation effect in synchrony, and thus cause a transient peak in population activity.

The negative transient in the population activity was a subsequent network effect of the positive transient: the synchronized spiking of the neurons participating in the positive transient was sufficient to drive a dip in activity in their target neurons in the other population.

### 4.1. Two forms of selection competition

Having established the existence and mechanics of the transient selection phenomenon, we sought to understand the conditions under which our striatal model could also support a steady-state competition effect, akin to classical winner-takes-all (Hartline and Ratliff, 1958; Fukai and Tanaka, 1997; Mao and Massaquoi, 2007). Such steady-state competition could plausibly arise in striatum as each projection neuron receives sufficient weak synapses from other projection neurons to continuously modulate its ongoing activity (Guzman et al., 2003; Humphries et al., 2010; Chuhma et al., 2011).

We found that increasing the number of projection neuron synapses gave rise to steady-state competition where the stable increase in activity in one population caused a stable decrease in activity of the other population. These results are consistent with Yim et al. (2011) who reported a weakly-competitive effect between two populations of neurons in a randomly-connected inhibitory network of spiking neurons, and showed that weak correlation between inputs to the network could enhance this effect. We advanced this result by showing that such steady-state competition could arise in both distance-dependent and randomly-connected networks, given either that we increased the physical size of our three-dimensional striatal network, and thus increase the density of connections, or randomly-connected the network based on the average connections of the most densely connected projection neuron.

Our models thus predict that the form of selection competition is dependent on the density of connections between projection neurons. Whether the striatum is ever as sparsely connected as in our distance-dependent model, or ever as densely connected as in the homogenous random model is an open question. It is possible that both forms of selection exist depending on local inhomogeneities in striatal tissue. We know that many aspects of the striatum shows gradients of density across the network, including the dorsal-ventral gradient of interneuron populations (Kubota and Kawaguchi, 1993) and the rostro-caudal gradient of FSI gap junctions (Fukuda, 2009). Correspondingly, it is plausible that there exists a gradient of projection neuron connection density.

We also note that the recent report by Oorschot et al. (2013) of projection neuron collaterals making synapses on to the somas of other projection neurons can only enhance both forms of competition. Such GABAergic somatic synapses are likely to shunt all dendritic input to the soma, thus providing powerful feedback inhibition. For transient selection, this could result in a larger negative transient; for steady-state selection, this could result in more depressed activity in the losing population. Open questions here include the relative density of such somatic synapses originating from projection neurons, and whether they have specific functional targets such as specifically occurring between projection neurons in competing populations.

Both forms of striatal selection mechanisms ultimately influence selection mediated by the whole basal ganglia network and expressed via their output nuclei (including SNr). As discussed in the Materials and Methods, this expression is via disinhibition (Chevalier and Deniau, 1990; Berns and Sejnowski, 1998; Redgrave et al., 1999; Gurney et al., 2001a; Humphries et al., 2006); increased activity of a striatal population inhibits the tonic inhibitory output of a SNr population, thus representing the selection of their represented signal (Figure 3). We showed that transient selection in the striatal populations is sufficient to enhance selection by disinhibition from SNr (Figure 6). This occurs because the most salient input causes a transient increase of activity in the corresponding striatal population and consequently transiently decreases the output of the corresponding SNr population. This fall is sufficient to allow activity to grow in the target thalamo-cortical loop, which in turn projects to the original striatal population, further increasing its activity—thus the positive feedback loop amplifies the transient changes in striatum. The effect of steady-state selection in the striatum on the whole basal ganglia is more straightforward. The long-lasting drop in output of all losing striatal populations comparatively reduces their inhibition of the corresponding SNr populations. Consequently, the fall in output of the SNr population representing the winning signal is enhanced compared to its competitors.

### 4.2. Experimental predictions of transient selection

Direct experimental observation of transient selection is challenging. The positive transient in population activity could only be observed on a single trial given sufficient simultaneous sampling of neurons within that population, a situation unlikely to occur with current recording technology. However, we showed that the basic mechanism underlying the positive transient in the population activity could be observed through sequential steps of current injection into a single neuron model. Thus our model makes a tractable experimental prediction: that there exists a regime of long, sequential steps of current into the projection neuron soma that will elicit a rapid burst of two or more spikes followed by slower regular firing. If such a regime exists, it would provide evidence in favor of the existence of transient selection mechanisms in the striatal network.

### 4.3. Transient selection alone could explain enhanced selection in huntington's disease

We sought to determine whether transient and steady-state selection could be differentiated by their predictions for how changes to the striatal circuit would affect selection. To this end, we asked if Huntington's-like changes of increased NMDA receptor sensitivity and loss of projection neurons could account for Beste et al. (2008)'s report of enhanced selection by Huntington's disease patients. In terms of our models, we asked if either transient or steady-state selection would improve due to these Huntington's-like changes to the striatum.

As one might expect *a priori*, simply removing projection neurons and thus reducing connectivity between them impaired both types of selection. Increasing NMDA receptor sensitivity also impaired steady-state selection, and thus this form of selection predicted that all Huntington's-like changes impair selection, a result which is inconsistent with the report by Beste et al. (2008). Surprisingly, however, we found that for transient selection, increased NMDA receptor sensitivity could more than compensate for cell loss and actually enhance selection. We also found that transient selectivity was only clearly improved with both high cell degradation and increased excitability, and thus not in pre-symptomatic-like conditions. Thus, alteration of transient selection and not steady-state selection in striatum is consistent with enhanced performance of symptomatic Huntington's disease patients compared to controls and pre-symptomatic patients.

Beste et al. (2008) noted that this enhanced response selection was paradoxical, as Huntington's disease patients are consistently worse than age-matched controls across a range of cognitive decision-making tasks (Knopman and Nissen, 1991; Bamford et al., 1995; Lawrence et al., 1998; Ho et al., 2003). Our models offer two potential explanations for why Huntington's disease related changes in striatum are usually associated with cognitive impairment but could also lead to paradoxical cognitive enhancement. First, suppose that all regions of striatum engaged by cognitive tasks implement transient selection. Our model shows that there are limited combinations of NMDA receptor sensitivity increase and cell atrophy where transient selection is enhanced compared to the healthy case; for most combinations transient selection is deteriorated compared to the healthy-state. Thus, one hypothesis is that there is a continuum of NMDA receptor sensitivity increase and cell atrophy across the striatum, and the Beste et al. (2008) task engaged a region of striatum with enhanced transient selection, whereas most tasks engage regions of the striatum with deteriorated transient selection. Second, suppose instead that different regions of striatum use transient or steady-state selection dependent on the local density of projection neuron connections. Our models shows that steady-state selection is always deteriorated by any Huntington's-like change to the striatum. Consequently, this suggests the hypothesis that the Beste et al. (2008) task engaged a region of the striatum using (enhanced) transient selection, whereas most cognitive tasks engage a region of striatum using steady-state selection, and thus are always deteriorated in Huntington's disease patients compared to the healthy-state.

## Funding

L'Agence Nationale de Recherche “NEUROBOT” project and a MRC Senior non-Clinical Fellowship (Mark D. Humphries); the EU Framework 7 “IM-CLeVeR” project (Kevin Gurney); EPSRC Green Brain project EP/J019534/1 (Eleni Vasilaki); EPSRC DTA student scholarship (Adam Tomkins); and Deutsche Forschungsgemeinschaft (DFG) Grant BE4045/10-1.

### Conflict of interest statement

The authors declare that the research was conducted in the absence of any commercial or financial relationships that could be construed as a potential conflict of interest.
